# Neutrophil Extracellular Traps and Endothelial Dysfunction in Atherosclerosis and Thrombosis

**DOI:** 10.3389/fimmu.2017.00928

**Published:** 2017-08-07

**Authors:** Haozhe Qi, Shuofei Yang, Lan Zhang

**Affiliations:** ^1^Department of Vascular Surgery, Renji Hospital, School of Medicine, Shanghai Jiao Tong University, Shanghai, China

**Keywords:** neutrophil extracellular traps, endothelial dysfunction, atherosclerosis, atherothrombosis, venous thromboembolism

## Abstract

Cardiovascular diseases are a leading cause of mortality and morbidity worldwide. Neutrophils are a component of the innate immune system which protect against pathogen invasion; however, the contribution of neutrophils to cardiovascular disease has been underestimated, despite infiltration of leukocyte subsets being a known driving force of atherosclerosis and thrombosis. In addition to their function as phagocytes, neutrophils can release their extracellular chromatin, nuclear protein, and serine proteases to form net-like fiber structures, termed neutrophil extracellular traps (NETs). NETs can entrap pathogens, induce endothelial activation, and trigger coagulation, and have been detected in atherosclerotic and thrombotic lesions in both humans and mice. Moreover, NETs can induce endothelial dysfunction and trigger proinflammatory immune responses. Overall, current data indicate that NETs are not only present in plaques and thrombi but also have causative roles in triggering formation of atherosclerotic plaques and venous thrombi. This review is focused on published findings regarding NET-associated endothelial dysfunction during atherosclerosis, atherothrombosis, and venous thrombosis pathogenesis. The NET structure is a novel discovery that will find its appropriate place in our new understanding of cardiovascular disease. In addition, NETs have high potential to be further explored toward much better treatment of atherosclerosis and venous thromboembolism in clinic.

## Highlights

1. The NET structure, which has been clearly observed in the atherosclerotic plaque and venous thrombi, is a novel discovery that will find its appropriate place in our new understanding of cardiovascular disease.
2. This review summarizes the crosstalk mechanism between NETs and endothelial cells during the thrombosis and atherosclerosis.
3. NETs have high potential to be further explored to progress toward much better treatment of atherosclerosis and venous thromboembolism in clinic.

## Introduction

Polymorphonuclear neutrophils (PMNs) have a significant innate immune system function in protection against pathogen invasion. In addition to classical phagocytosis, PMNs can release chromatin, nuclear proteins, and serine proteases extracellularly to form [neutrophil extracellular traps (NETs)], which comprise net-like DNA fibers containing histones and antimicrobial proteins (1). NETs can entrap pathogens to limit their dispersion, trigger coagulation, and induce endothelial injury. Since the first characterization of NETs in 2004, studies of their effects have expanded to reveal unexpected roles in sterile inflammation induced by PMNs (2, 3). Notably, the antibacterial activity of NETs is abrogated by deoxyribonuclease (DNase), which can directly degrade the chromatin fibers that comprise the backbone of NETs (2). Vascular endothelial cells (ECs) maintain the balance between anticoagulation and immune response functions. Atherosclerosis and venous thromboembolism (VTE) are two major cardiovascular diseases associated with endothelial dysfunction. Atherosclerosis and thrombosis share many common risk factors, such as obesity, diabetes, smoking, hypertension, and hyperlipidemia; however, it remains unclear whether there are specific factors involved in the pathogenesis of both atherosclerosis and VTE (4).

Neutrophil extracellular traps can be detected in both atherosclerosis and thrombosis, and the existence of these structures could be perceived as a double-edged sword in the context of disease processes, as it may both attenuate tissue injury and amplify local inflammation, leading to deterioration in disease symptoms (5). Nevertheless, no specific explanations are available for the effects of NETs on vascular endothelial function and the promotion of atherosclerosis and thrombosis. In this review, we reveal potential mechanisms underlying NET formation and endothelial dysfunction in cardiovascular disease and examine current knowledge of the potential clinical implications of these structures.

## Mechanism of NET Formation

Neutrophil extracellular traps are formed during inflammation and observed *in vivo* during infections (6). The existence of NETs indicates that PMNs may undergo an alternative form of programmed cell death, termed NETosis, allowing function of these structures in innate immune defense. Depending on the different triggers involved, signaling molecule receptors and membrane integrity, NETosis is described as either “vital” or “suicidal” (7–11). In “vital” NETosis, PMNs rapidly release nuclear DNA encircled by vesicles to the extracellular space without membrane perforation, in response to stimulation by platelets *via* toll-like receptor (TLR)-4, or Gram-positive bacteria *via* TLR-2, in a reactive oxygen species (ROS)-independent manner (12). “Suicidal” NETosis is characterized by strong activation of nicotinamide adenine dinucleotide phosphate oxidase by phorbol 12-myristate 13-acetate, interleukin-8 (IL-8), or various microbial pathogens, in a ROS-dependent manner (13, 14). NETs can be released *via* neutrophil lysis or through vesicular transport of nuclear or mitochondrial DNA, without membrane rupture (12, 15). Regardless of which type of NET occurs, the molecular contents of their structures are similar, and include histones, neutrophil elastase (NE), myeloperoxidase (MPO), proteinase 3, cathepsin, and gelatinase (16, 17).

Although neutrophils are transcriptionally active cells, the majority of their DNA is transcriptionally inactive and condensed into heterochromatin. Its decondensation is mediated by peptidyl arginine deiminase 4 (PAD4), which catalyzes the conversion of histone arginines to citrullines, reducing the strong positive charge of histones, and consequently weakening histone-DNA binding (18). Spikes in intracellular Ca^2+^ can activate PAD4 to propagate NET release, and PAD4-deficient mice are unable to form NETs in response to physiological activators, such as bacteria (19, 20). NE is considered essential for histone cleavage during NETosis; accordingly, secretory leukocyte peptidase inhibitor, an endogenous elastase inhibitor, can inhibit NETosis (14, 21). The central role of elastase in NETosis is corroborated by the inability of PMNs from elastase-deficient mice to undergo this process (22).

## NETs and Atherosclerosis

**Figure 1:**
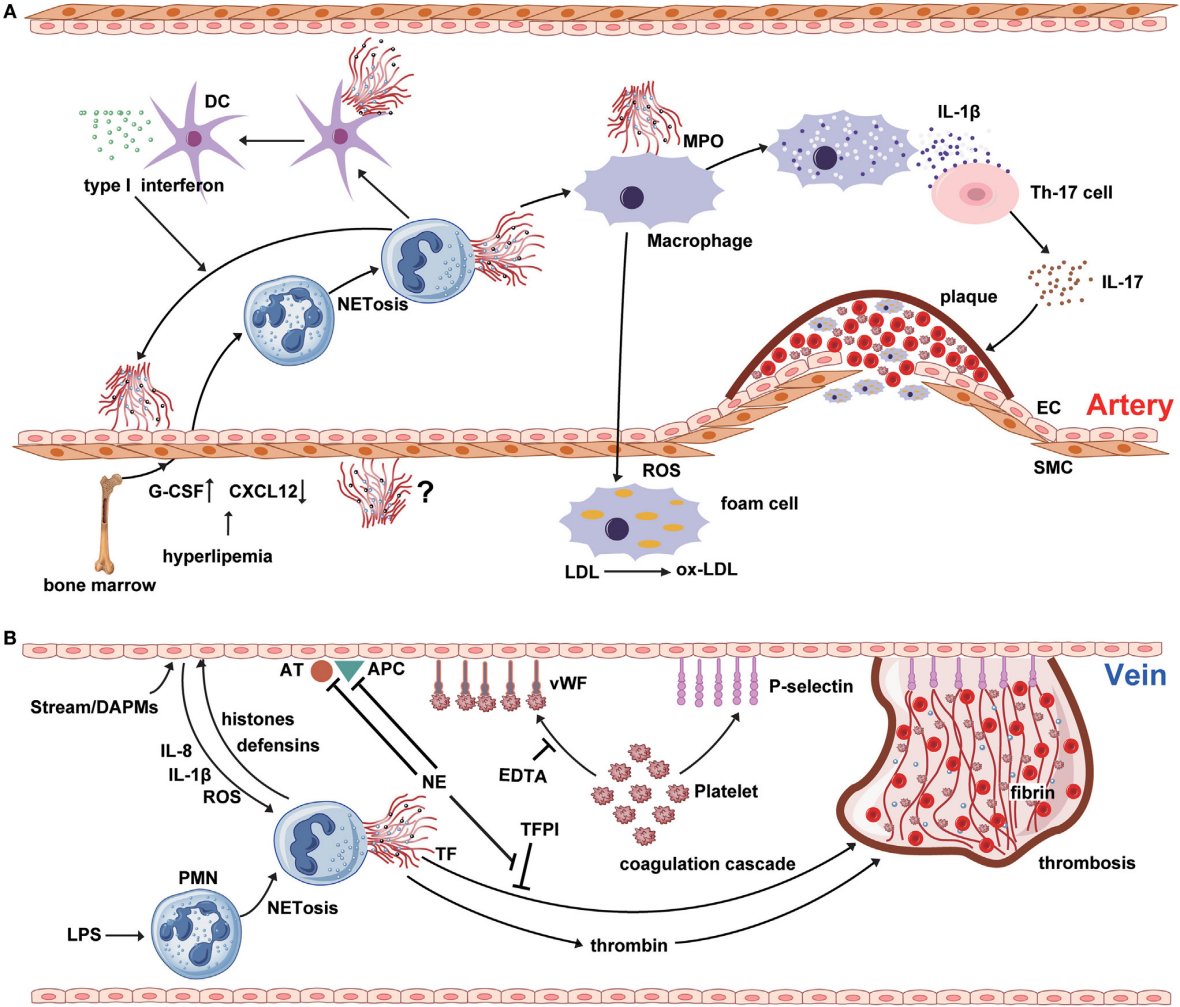
NETosis interweaves atherosclerosis and thrombosis. **(A)** Neutrophil extracellular traps (NETs) are involved in the whole process of atherosclerosis. The myeloperoxidase from NTEs can stimulate macrophage to oxidize low-density lipoprotein (LDL) to ox-LDL and form the foam cell. The hyperlipidemia recruits neutrophil into circulation from bone marrow by upregulating the expression of granulocyte colony-stimulating factor and downregulating the level of C-X-C motif ligand -12, which is an important signal for the clearance and recruitment of aged neutrophils to the bone marrow. Cholesterol crystals can trigger the polymorphonuclear neutrophil (PMN) to release the NETs that prime the macrophages for pro-inflammatory cytokine production including IL-1β. Then IL-1β activates Th17 cell to release interleukin-17, amplifying the immune cell recruitment into the atherosclerotic plaque. As another critical source of foam cell, SMC also takes part in atherosclerosis. However, there are few reports about the interaction between NETs and SMC. **(B)** NETs are released from PMNs, which are activated by LPS or other cytokines from injured endothelial cells. NETs promote the expression of von Willebrand factor and P-selectin on the surface of venous endothelium to entrap both platelets and red blood cells, thereby creating a scaffold for fibrin deposition. Meanwhile, histones and TF from the NETs structure induce the thrombin generation and activation *via* platelet-dependent or -independent mechanism. Tissue factor pathway inhibitor (TFPI) can abrogate the function of TF. However, utrophil elastase from NETs could degrade TFPI, antithrombin, and activated protein C.

Atherosclerosis is a cardiovascular disease accompanied by chronic vascular wall inflammation, endothelial dysfunction, and smooth muscle cell proliferation (23). Given the limited lifespan of PMNs and inadequate methods for their detection, the contribution of neutrophils to atherosclerosis has been underestimated (24). Additionally, the phenotype of PMNs can alter in response to inflammation, which has also resulted in the historical neglect of the role of neutrophils in the process of atherosclerosis (Figure 1A) (25). Hyperlipidemia can injure ECs, promoting lipid deposition and plaque formation, and usually represents the onset of atherosclerosis. Interestingly, hyperlipidemia induces neutrophilia, which is positively associated with atherosclerotic plaque burden (24). In addition, hypercholesterolemia can induce the synthesis of granulocyte colony-stimulating factor (G-CSF), a key cytokine in the regulation of granulopoiesis, through inducing increased levels of tumor necrosis factor-α and interleukin-17 (IL-17) (26). G-CSF stimulates the proliferation of myeloid precursors and reduces bone marrow C-X-C motif ligand (CXCL)-12 levels, thereby reducing the clearance of aged PMNs (27). In addition, hypercholesterolemia can enhance serum levels of CXCL1, which promotes PMN mobilization (28). Together, these data suggest that PMNs may play a role in stimulation of atherosclerosis.

Recent studies have indicated that PMNs attach themselves to atherosclerotic plaques, primarily through NET formation (Figure 1A). Components of NETs, such as cathepsin G and cathelicidins, exhibit monocyte-attracting activity in atherosclerotic plaques (29, 30). The cathelicidin-related antimicrobial peptide (CRAMP) residing in neutrophil secondary granules have potent effects on recruitment and activation of immune cells, such as monocytes and dendritic cells (31). NET-derived CRAMP-deleted ApoE-deficient mice develop smaller plaques than ApoE-deficient mice, suggesting that CRAMP may be involved in plaque formation (32). Moreover, NETs have been identified as a major source of CRAMP, which is deposited directly on the inflamed endothelial surface in atherosclerotic vessels. Indeed, NET-derived CRAMP anchors to ECs, where it can link with formyl-peptide receptor 2 on classical monocytes, resulting in monocyte recruitment to ECs (33). After binding to the mannose receptor of macrophages, MPO from NETs induces the release of ROS, along with other pro-inflammatory macrophage-derived cytokines (23). Furthermore, proteinases from NETs affect plaque instability, while ROS from macrophages contributes to the modification of low-density lipoprotein (LDL) to produce ox-LDL, which promotes the development of foam cells (34, 35). NETs can also regulate cytokine production from macrophages in atherosclerosis (36). More precisely, cholesterol crystals function as danger signals, inducing interleukin-1β (IL-1β) production and triggering NET release from PMNs. Subsequently, NETs stimulate cytokine release from macrophages and activate T helper 17 cells, resulting in amplified immune cell recruitment to the atherosclerotic plaque (36).

Research has underscored the importance of NETs in the regulation of lesion size in atherosclerosis, and suggests that these structures can induce endothelial dysfunction directly by activation and damage of ECs (37). Inhibition of PAD4 using chloramidine led to decreased atherosclerotic lesion size and carotid artery thrombosis delay in a mouse model, while these effects were not observed after treatment with neutrophil-depleting antibody, or of mice lacking a functional type I interferon receptor (37). These data indicate a critical direct role for NETs in atherosclerotic lesion formation *via* type I interferon. Mixture of cell free-DNA and granule proteins can stimulate plasmacytoid dendritic cells, leading to a strong type I interferon response and a deteriorating atherosclerotic plaque burden; however, the importance of the NET-derived type I interferon response in atherogenesis has been questioned, because NETs can also regulate cytokine production by macrophages in atherosclerosis (36, 38, 39).

## NETs and Atherothrombosis

Atherothrombosis is the formation of a thrombus within an artery with atherosclerosis. Neutrophils as well as macrophages participate importantly in this disease process. In most cases, atherothrombosis follows rupture of atheroma, which may be triggered by NETs (8). Circulating leukocytes have a crucial role in atherothrombosis and systemic neutrophil counts are robust predictors of acute coronary events (40, 41). Moreover, complement activation can trigger PMN recruitment to the site of atherothrombosis in acute myocardial infarction (42). Coronary atherothrombosis specimens from patients with acute myocardial infarction contain numerous activated neutrophils (43–45). Sudden rupture of atherosclerotic plaques triggers platelet aggregation and fibrin deposition at the initial site of atherothrombosis to entrap circulating red blood cells. The interaction of thrombin-activated platelets with PMNs at the site of plaque rupture during acute ST-segment elevation acute myocardial infarction results in local formation of NETs (46). Elevated levels of circulating DNA and chromatin released from activated PMNs are independently associated with severe coronary atherosclerosis and the prothrombotic state (47). Interestingly, NETs are frequently found in lytic and fresh thrombus specimens, but never observed in organized thrombus (48). Hence, it can be assumed that NETs are involved at an early stage during the formation of coronary thrombus and lytic changes. A recent study involving evaluation of coronary atherothrombosis specimens demonstrated that NET burden and DNase activity in ST-elevation acute coronary syndrome are predictors of ST-segment resolution and infarct size (49).

Histological analysis of 26 thrombectomy samples from patients with acute myocardial infarction revealed that activated platelets present high-mobility group box 1 protein to PMNs, thereby inducing NET formation (50). The authors speculate that these NETs may contribute to plaque rupture and subsequent thrombus formation. In accordance with these findings, platelet-derived high-mobility group box 1 protein can facilitate NET formation and coagulation (51). Similarly, Riegger et al. analyzed 253 samples from patients with stent thrombosis after percutaneous coronary intervention (52). Approximately 23% of the thrombi specimens contained NETs; however, no differences in the number of NETs were observed according to the timing of stent thrombosis, stent type, or in comparison with samples from patients with spontaneous myocardial infarction (52). Hence, recruitment of PMNs appears to be a hallmark of stent thrombosis.

As the main initiator of coagulation, with a critical role in arterial thrombosis, tissue factor (TF) has been investigated in patients with acute ST-segment-elevation myocardial infarction (53). Local accumulation of TF-bearing NETs is observed at sites of coronary thrombosis, and PMNs release NETs, thereby exposing TF in infarct-associated, but not non-infarcted, areas (53). In addition, neutrophil islets and NETs decorated with TF were detected in thrombi obtained from infarcted regions (46). Interactions between activated platelets and PMNs at sites of plaque rupture during acute myocardial infarction result in NET formation and delivery of active TF, which together foster thrombus formation. Notably, NETs were also identified as coated with IL-17, which promotes thrombosis by enhancing platelet aggregation in coronary thrombectomy samples (48). The role of NETs has also been examined in a model of myocardial ischemia–reperfusion, and a significant cardioprotective effect of NET-inhibition treatment on myocardial ischemia–reperfusion injury was clearly demonstrated (54).

## NETs and VTE

Deep venous thrombosis (DVT) and pulmonary embolism are designated ‘VTE’ in the clinic. Venous thrombogenesis is usually accompanied by inflammatory reactions of ECs (55). As a key element of the inflammatory response, NETs also play an important role in venous thrombogenesis (Figure 1B). Unlike atherothrombosis, the onset of venous thrombosis is primarily initiated by endothelial injury, caused by disturbance of the blood stream or endothelial dysfunction, and mediated *via* damage-associated molecular patterns (56). Subsequently, Weibel–Palade bodies derived from ECs secrete massive amounts of von Willebrand factor (vWF) and P-selectin, which adhere to platelets and recruit leukocytes (57, 58). NETs predominantly form during the organizing stage of human VTE development (59). At the local lesion site, platelets interact directly with PMNs and promote the production of NETs (60). Additionally, cytokines from activated ECs (e.g., IL-1β, IL-8, and ROS) can accelerate NET formation (61). NETs, in turn, induce EC activation through NET-derived proteases; for example, histones and defensins (62). Additionally, purified histones can enhance thrombin generation through both platelet-dependent and platelet-independent mechanisms; however, platelet aggregation in response to histone H3 is inhibited by ethylenediaminetetraacetic acid (EDTA), suggesting that platelet aggregation is caused by the positive charge of histones (63–65). Intravenous administration of exogenous histones accelerates clot formation, whereas DNase treatment significantly delays the onset of DVT (66). In addition to clinical investigations, studies in mice have identified an association between the risk of DVT and high PMN counts, supporting an important and early role for NETs in venous thrombosis (67, 68).

In the process of thrombosis propagation, circulating nucleosomes act as a platform for the degradation of tissue factor pathway inhibitor, which is mediated by NE (69). The levels of circulating nucleosomes in DVT patients are significantly elevated, and may be a useful plasma marker for NET formation (70). In addition to providing an adhesive platform for platelets, NETs also support the adhesion of red blood cells (65). NETs maintain the stability of thrombus *via* vWF, fibronectin, and fibrinogen; vWF and fibrinogen can interact with histones, and fibronectin has a DNA-binding domain (71). Heparin can remove histones, leading to the destabilization of NETs (72). *In vitro* data support the ability of NETs to stimulate the activation of coagulation cascades and platelet adhesion, and fibrin deposition colocalizes with NETs in blood clots (69). Purified histones impair thrombomodulin-dependent protein C activation to enhance plasma thrombin generation (73). Furthermore, DNA and histones interact with and trap platelets, most likely *via* electrostatic interactions or TLRs (73). Together, the findings described above indicate that NETs make a substantial contribution to maintenance of the stability of venous thrombi.

Monocytes are recruited during thrombosis and thrombolysis; however, the specific function of monocytes in dissolving NET-induced thrombus requires further investigation. NETs colocalize with fibrins and vWF in venous thrombi, and vWF and fibrins constitute the main scaffold that must be fragmented in order to destroy the integrity of the thrombus structure (Figure 1B) (65, 66). *In vitro*, NETs can provide a scaffold for clots to induce resistance to tPA-induced thrombolysis (65). DNase is a strong nuclease present in blood and has the power to degrade protein-free DNA; however, the ability of DNase to degrade NET-derived chromatin is limited, because their chromatin is decorated with numerous proteases and histones. Interestingly, DNase can cooperate with the plasminogen system during chromatin degradation (74). In addition, NETs may recruit plasminogen from the plasma. Histone H2B can serve as a receptor for plasminogen on the surface of human monocytes/macrophages and could potentially also serve this function in NETs (75). *In vitro* studies have shown that NET-derived NE and cathepsin G can degrade fibrin and enhance fibrinolysis in DVT (76). Plasma DNA concentrations correlate with D-dimer levels; therefore, it is plausible that circulating DNA may reflect the degradation of NETs within a thrombus (70, 77).

In immunothrombosis, NETs may function in capture of invasive pathogens, prevention of distant tissue involvement, concentration of pathogens for bactericidal killing, and recruitment of other immune cells to immune target sites (78). In models of sepsis, lipopolysaccharide can activate platelets and PMNs *via* TLR-4 to induce NETosis (50, 79). We have reported that PMNs from septic patients have significantly enhanced NET release, compared with those from healthy controls with increased risk of VTE (80). NET-associated immunothrombosis leads to more sturdy thrombi with reduced permeability and decreased susceptibility to thrombolysis, although this can be overcome with DNase treatment (81). In addition to sepsis, NETs and immunothrombosis have been implicated in other autoimmune diseases, including inflammatory bowel disease and vasculitis (82, 83).

## Future Challenges and Clinical Implications

Undoubtedly, more in-depth studies are needed to meticulously dissect the exact mechanisms of *in vivo* NET formation, and to clarify the importance of histone citrullination for NETosis (84). ROS generation by different types of leukocyte is a common trigger of NETosis; however, the exact mechanism of ROS-induced NETs formation and subsequent endothelial dysfunction is unclear (Figure 1). Moreover, how NET-derived proteases respond in atherosclerosis and thrombosis remains an open question. Movement from investigations of integrated NETs to study of more specific *in vitro* protease systems, which may better explain the phenomena associated with disease, is an interesting future prospect. NETs have been identified at each stage of cardiovascular disease. Nevertheless, whether NETs play different roles at different stages remains unknown. Additionally, it will be a challenge to explore whether NETs are involved in cross talk with smooth muscle cells, which are another major source of foam cells during atherosclerosis. Regarding DVT, it will be important to identify endogenous triggers of NET formation. Furthermore, whether the NETs involved in DVT are generated by cell lysis or a secretory process is another a critical question. A better understanding of NETosis, both with regards to structural constituents and context-specific functional decoration, will be a prerequisite to further elucidation of the role of NETs in atherosclerotic plaques and venous thrombus, and will be of paramount importance to the identification, validation, and implementation of the best molecular candidates for therapeutic targeting.

The notion that NETs represent a mechanism by which PMNs release thrombogenic signals during atherosclerosis and thrombosis may offer novel therapeutic targets (Table 1). Thrombolysis has become a key weapon in the arsenal against pathologic thrombosis; however, not all thrombotic events are susceptible to thrombolysis. Indeed, the addition of DNA and histones to a fibrin matrix has been shown to generate artificial thrombus more resistant to tissue plasminogen activator, and which can be partially remedied by DNase (85). Preliminary data from murine models of DVT demonstrate inhibition of thrombus formation by DNase treatment prior to model establishment (66, 67). Although DNase treatment, which likely enhances thrombolysis, appears to harbor relevant therapeutic potential, its utility and applicability to prevention of NET formation or digestion of established NETs to reduce atherosclerotic lesion growth is debatable and will remain controversial (86). Moreover, knockout of neutrophil oxidase 2, a NET component, can result in accelerating disease in a murine model of lupus; therefore, caution is required in the selection of NET-associated molecular targets. Another potential target is NET-related platelet recruitment to the endothelium (37). Specifically, blockade of platelet alpha-granule or endothelial Weibel–Palade body release would decrease P-selectin- and vWF-mediated platelet and PMN recruitment to the endothelium, thereby decreasing NETosis (87). Similarly, vWF degradation enzyme could be administered to prevent PMN recruitment with subsequent NETosis (88). Although these countermeasures may result in mild immunodeficiency, they could also abrogate pathologic immune-mediated thrombosis without sacrificing immune competence when administered in a controlled manner. It is noteworthy that NETs are not major role players in these diseases but may definitely exacerbate the condition and therapies may have to be combinatorial because NET formation is only one of the factors.

**Table 1:** Potential targets for translation in the prevention of NET-mediated atherosclerosis and thrombosis.

Diseases	Target	Function	Clinical implications	
			Clinical therapies	Potential treatment
Atherosclerosis	cfDNA	Stimulation of plasmacytoid dendritic cells		Deoxyribonuclease (86)
	MPO	Induction of ROS		Ocimum tenuiflorum (89)
	Cathepsin G	Attraction of monocyte		Ac-Phe-Val-Thr-(4-guanidine)-Phg(P)-(OPh4-SMe)2 (90)
	Cathelicidins	Attraction of monocyte		
	IL17	Amplification of platelet aggregation		
	IL-1β	Amplification of inflammatory reaction; stimulation to Th17 cells	Rimonabant (91)	

Venous thromboembolism	Histones	Endothelial injury		Activated protein C (62)
	NE	Degradation of TFPI and fibrin		Leu89 with alanine (69)
	Cathepsin G	Degradation of fibrin and enhancement of fibrinolysis		Leu89 with alanine (69)
	P-selectin	Adherence to platelet		Anti-P-selectin aptamer; anti-P-selectin glycoprotein ligand-1 inhibitory antibody (58)
	vWF	Adherence to platelet		Anti-P-selectin glycoprotein ligand-1 inhibitory antibody (92)
	TF	Activation of coagulation cascade		TFPI (93)
	AT	Anticoagulation		
	APC	anticoagulation		
	Thrombin	Promotion of coagulation		PAR-1 antagonists (94)
	Fibrin	Promotion of coagulation	Urokinase; rtPA	

*Antimicrobial proteins (1)*.

*NETs, neutrophil extracelluar traps; cfDNA, cell-free DNA; MPO, myeloperoxidase; ROS, reactive oxygen species; IL-17, interleukin 17; IL-1β, interleukin 1β; NE, neutrophil elastase; TFPI, tissue factor pathway inhibitor; vWF, von-Willebrand factor; TF, tissue factor; AT, antithrombin; PAR, proteinase-activated receptor; APC, activated protein C; rtPA, recombinant tissue plasminogen activator*.

## Conclusion

Neutrophil extracellular trap-structure is an important novel discovery that has potential to influence our understanding of cardiovascular disease. Functionally, NETs can induce activation of ECs, antigen-presenting cells, and platelets, and cause endothelial dysfunction, resulting in a proinflammatory immune response. As evidenced by the results of the studies discussed above, NETs can clearly contribute to the initiation and progression of atherosclerotic and thrombotic lesions. Moreover, there is evidence for an emerging role of PMNs, focused on NETosis and oxidative stress burden, in orchestrating common mechanisms involved in various forms of cardiovascular disease. Extensive future research will be required to determine the effects of NETs in endothelial dysfunction-induced cardiovascular disease; hence, the time is not yet ideal to implement therapeutic options targeting neutrophils in the context of atherosclerosis and thrombosis.

## Author Contributions

HQ contributed to the conception of the study, consulting literatures, and manuscript preparation; SY make the figure and modify the manuscript; LZ helped perform the analysis with constructive discussions.

## Conflict of Interest Statement

The authors declare that the research was conducted in the absence of any commercial or financial relationships that could be construed as a potential conflict of interest.

## Abbreviations

NETs, neutrophil extracellular traps; PMN, polymorphonuclear neutrophil; DNase, deoxyribonuclease; ECs, endothelial cells; VTE, venous thromboembolism; TLR, toll-like receptor; ROS, reactive oxygen species; NE, neutrophil elastase; PAD4, peptidyl arginine deiminase 4; G-CSF, granulocyte colony-stimulating factor; MPO, myeloperoxidase; IL, interleukin; CXCL, C-X-C motif ligand; LDL, low-density lipoprotein; DVT, deep venous thrombosis; PE, pulmonary embolism; vWF, von Willebrand factor; EDTA, ethylenediamine-tetraacetic acid.
